# Genome Sequence of *Streptomyces wadayamensis* Strain A23, an Endophytic Actinobacterium from *Citrus reticulata*

**DOI:** 10.1128/genomeA.00625-14

**Published:** 2014-07-03

**Authors:** Luciana G. de Oliveira, Gabriela D. Tormet Gonzalez, Markyian Samborsky, Joelma Marcon, Welington L. Araujo, João Lucio de Azevedo

**Affiliations:** a Department of Organic Chemistry, University of Campinas, UNICAMP, Campinas, São Paulo, Brazil; b Department of Biochemistry, University of Cambridge, Cambridge, England; c Department of Genetics, Luiz de Queiroz College of Agriculture, University of São Paulo, Piracicaba, São Paulo, Brazil; d Department of Microbiology, Institute of Biomedical Sciences, University of São Paulo, São Paulo, Brazil

## Abstract

The actinobacterium *Streptomyces wadayamensis* A23 is an endophyte of *Citrus reticulata* that produces the antimycin and mannopeptimycin antibiotics, among others. The strain has the capability to inhibit *Xylella fastidiosa* growth. The draft genome of *S. wadayamensis* A23 has ~7.0 Mb and 6,006 protein-coding sequences, with a 73.5% G+C content.

## GENOME ANNOUNCEMENT

*Streptomyces wadayamensis* strain A23 is an actinobacterium isolated from the population existing in the plant tissue of *Citrus reticulata* (tangerine) (1). The isolate shows a high capability to promote *in vitro* inhibition of *Xylella fastidiosa* growth, as well as to promote the degradation of xanthan gum. Additionally, ethyl acetate extracts from *S. wadayamensis* fermentation were able to inhibit the *Candida albicans* pathogen, *Neisseria meningitidis* strains, and multiresistant *Staphylococcus aureus* (L. G. de Oliveira, P. L. R. Cruz, A. B. Gonçalves, M. Samborsky, A. F. Vivian, E. M. Schmidt, M. N. Eberlin, and W. L. Araújo, unpublished data).

The draft genome sequence of *S. wadayamensis* was generated using Illumina MiSeq technology at the Department of Biochemistry, University of Cambridge, Cambridge, England, supported by the São Paulo Funding Agency. The libraries of the *S. wadayamensis* strain were prepared using the Nextera library prep kit, sequenced using V2 Illumina sequencing chemistry, and run on MiSeq (2 × 150 bp paired-end [PE] sequencing). The Illumina shotgun library produced 7.8 million reads in a total of 2.4 Gb data. After filtering and adapter and quality trimming, the sequencing results were assembled using the Lasergene SeqMan NGen version 3.1 (DNAStar) assembler program, providing 96× total genome coverage. The assembly was converted to Consed-compatible ace format and checked using Consed (2, 3), generating 180 contigs. Annotation was carried out using a customized pipeline based on FgeneSB, operating in the *ab initio* mode. The annotation results were edited using Artemis (4). RAST server annotation (5) allowed the identification of 6,006 candidate protein-coding genes. Additionally, 17 transcription factor initiators and subsystems related to the biosynthesis of secondary metabolites, fatty acids, lipids, isoprenoids, and siderophores, such as desferrioxamine and aerobactin, were identified. The total genome size comprises 7,056,544 bp, with a G+C content of 73.5%.

It is widely known that members of the *Streptomyces* genus carry a very versatile group of biosynthetic machineries capable of producing complex molecules that present antibiotic, antitumor, and immunosuppressant activities. Careful analysis of the genome annotation revealed a great ability to biosynthesize antibiotic metabolites. Further analysis through antiSMASH (6) revealed that the *S. wadayamensis* A23 genome contains at least 36 gene clusters coding for the biosynthesis of t1-polyketide synthase (PKS), t2-PKS, and t3-PKS, nonribosomal peptide synthase (NRPS), terpene, lantipeptide, siderophore, bacteriocin, and ectoine and NRPS-t1-PKS, lantipeptide-NRPS-t1-PKS, tiopeptide-lantipeptide, and bacteriocin-terpene mixed clusters. Siderophores, such as aerobactin, enterobactin, and desferrioxamine B and E, nonribosomal peptides, such as mannopeptimycin, and NRPS-PKS antimycins A1, A4, A7, and A8 are among the metabolites already identified from culture extracts.

Though no xanthanases or xanthan degradation-specific enzymes were suggested in the genome annotation, several enzymes involved in mannose metabolism, alpha- and beta-glucosidases, and secreted endoglucanases were annotated, suggesting that fastidian gum might be degraded by this set of enzymes, and anticipating that *S. wadayamensis* might assist in the control of *X. fastidiosa* pathogenicity in *C. reticulata*.

### Nucleotide sequence accession numbers.

This whole-genome shotgun project has been deposited at DDBJ/EMBL/GenBank under the accession no. JHDU00000000. The version described in this paper is the first version, JHDU01000000.

## References

[1] Araújo WL Marcon J Maccheroni W Jr van Elsas JD Van Vuurde JW Azevedo JL 2002 Diversity of endophytic bacterial populations and their interaction with *Xylella fastidiosa* in citrus plants. Appl. Environ. Microbiol. 68:4906–4914. 10.1128/AEM.68.10.4906-4914.2002 12324338 PMC126398

[2] Gordon D Green P 2013 Consed: a graphical editor for next-generation sequencing. Bioinformatics 29:2936–2937. 10.1093/bioinformatics/btt515 23995391 PMC3810858

[3] Gordon D Abajian C Green P 1998 Consed: a graphical tool for sequence finishing. Genome Res. 8:195–202. 10.1101/gr.8.3.195 9521923

[4] Rutherford K Parkhill J Crook J Horsnell T Rice P Rajandream MA Barrell B 2000 Artemis: sequence visualization and annotation. Bioinformatics 16:944–945. 10.1093/bioinformatics/16.10.944 11120685

[5] Aziz RK Bartels D Best AA DeJongh M Disz T Edwards RA Formsma K Gerdes S Glass EM Kubal M Meyer F Olsen GJ Olson R Osterman AL Overbeek RA McNeil LK Paarmann D Paczian T Parrello B Pusch GD Reich C Stevens R Vassieva O Vonstein V Wilke A Zagnitko O 2008 The RAST server: Rapid Annotations using Subsystems Technology. BMC Genomics 9:75. 10.1186/1471-2164-9-75 18261238 PMC2265698

[6] Blin K Medema MH Kazempour D Fischbach MA Breitling R Takano E Weber T 2013 antiSMASH 2.0—a versatile platform for genome mining of secondary metabolite producers. Nucleic Acids Res. 41:W204–W212. 10.1093/nar/gkt449 23737449 PMC3692088

